# Mechanisms responsible for postmenopausal hypertension in a rat model: Roles of the renal sympathetic nervous system and the renin–angiotensin system

**DOI:** 10.14814/phy2.12669

**Published:** 2016-01-26

**Authors:** Rodrigo O. Maranon, Jane F. Reckelhoff

**Affiliations:** ^1^ Women's Health Research CenterDepartment of Physiology and BiophysicsUniversity of Mississippi Medical CenterJacksonMississippi

**Keywords:** Aging, female, losartan, menopause, renal denervation

## Abstract

Hypertension in postmenopausal women is less well controlled than in age‐matched men. The aging female SHR is a model of postmenopausal hypertension that is mediated in part by activation of the renin–angiotensin system (RAS) and by the renal sympathetic nervous system. In this study, the hypothesis was tested that renal denervation would lower the blood pressure in old female SHR and would attenuate the antihypertensive effects of AT1 receptor antagonism. Retired breeder female SHR were subjected to right uninephrectomy (UNX) and left renal denervation (RD) or UNX and sham, and 2 weeks later, baseline mean arterial pressure (MAP; radiotelemetry) was measured for 4 days, and then rats were treated with angiotensin (AT1) receptor antagonist, losartan (40 mg/kg/day po) for 6 days. Renal denervation reduced MAP in old females compared to sham (172 ± 6 vs. 193 ± 6 mm Hg; *P* < 0.05). Losartan reduced MAP in both sham and RD rats similarly (numerically and by percentage) (142 ± 10 vs. 161 ± 6 mm Hg; *P* < 0.05 vs. RD,* P* < 0.05 vs. baseline). However, female SHR rats remained significantly hypertensive despite both pharmacological intervention and RD. The data suggest that both the renal sympathetic nervous system and the RAS have independent effects to control the blood pressure in old female SHR. Since the denervated rats treated with losartan remained hypertensive, the data also suggest that other mechanisms than the RAS and renal sympathetic nervous system contribute to the hypertension in old female SHR. The data also suggest that multiple mechanisms may mediate the elevated blood pressure in postmenopausal women.

## Introduction

Blood pressure is less well controlled in postmenopausal women than in age‐matched men (National Center for Health Statistics, Health, United States, 2011; Lima et al. 2012). The reasons for this gender difference are not clear since women likely consult their health care providers at least as often as men do and are likely to be as compliant as men in taking their medications. Perhaps, the mechanisms responsible for the hypertension are different between aging men and women, and this could account for the gender difference in hypertension control. The current guidelines for treatment of hypertension are not different for aging men and women (James et al. 2014); so, if there are gender differences in the mechanisms that mediate blood pressure control, these differences would not be acknowledged with specific treatment under the current guidelines.

In recent years, we have used the spontaneously hypertensive rat (SHR) as a model for postmenopausal hypertension (Fortepiani et al. 2003), and have evaluated the mechanisms responsible for their elevated blood pressure. The female SHR have lower blood pressure than males prior to 10–12 months of age when they stop estrous cycling (Fortepiani et al. 2003). By 16 months of age, the blood pressure in females is increased to similar or higher levels than the blood pressure in age‐matched male SHR (Fortepiani et al. 2003).

We have evaluated various systems known to control hypertension to determine the mechanisms responsible for the higher blood pressure in the old female SHR. For example, renal denervation reduced the blood pressure in old females, but the rats remained significantly hypertensive following denervation (Maranon et al. 2014), suggesting that other mechanisms also contributed to the control of the blood pressure in the old females. In another study, we evaluated the role of the renin–angiotensin system, and found that the angiotensin AT1 receptor antagonist, losartan, also reduced the blood pressure in old female SHR, but again the rats remained hypertensive (Yanes et al. 2010), again suggesting the contribution of other systems to mediate the hypertension in the old females.

Whether the renal sympathetic nerves and the RAS have independent or additive affects on blood pressure in the old female SHR is unknown. Activation of the renal sympathetic nervous system can cause renin release (Johns 2014). In an effort to control the total body volume, increases in renal sympathetic nerve activity can inhibit natriuresis and diuresis, resulting in lower levels of sodium and water reaching the macula densa and thus cause renin release (Johns 2014). Alternatively, a larger increase in renal sympathetic nerve activity could cause a reduction in renal blood flow and glomerular filtration rate, leading to greater sodium reabsorption and inhibit renin release to prevent further increases in blood pressure. For the present studies, the hypothesis was tested that renal denervation would lower the blood pressure in old female SHR, thus reducing renin release and angiotensin II synthesis, and thus would attenuate the antihypertensive effects of AT1 receptor antagonism.

## Methods

### Rats

Retired breeder female SHR were obtained from the vendor (Taconic Laboratories, Hudson, NY) at 4–9 months of age, and were allowed to age to 16 months of age in the Laboratory Animal Facility of the University of Mississippi Medical Center. Rats were maintained on standard laboratory chow (Teklad, Harlan Sprague Dawley, Indianapolis, IN) and tap water with 12 h:12 h light:dark cycle. All protocols were reviewed and approved by the Institutional Animal Care and Use Committee of the University of Mississippi Medical Center, and studies were performed in accordance with the *Guide for the Care and Use of Laboratory Animals*, 8th Edition, 2011, National Institutes of Health.

### Experimental design

Under gas anesthesia with isoflurane (Mallinckrodt Veterinary, Hazelwood, CA) and with aseptic technique, rats were subjected to removal of the right kidney and renal denervation (*n* = 5) or right nephrectomy and sham denervation (isolation of the renal nerves) (*n* = 4), as we previously described (Maranon et al. 2014). Uninephrectomy was performed in both groups of rats and does not further increase blood pressure in female SHR (Maranon et al. 2014) since they have very little renal injury at this age as noted by urinary protein excretion (Fortepiani et al. 2003). Rats were also implanted with radiotelemetry transmitters (TA11PA‐C40; Data Sciences International, St. Paul, MN) into the abdominal aorta below the renal arteries, as we previously described (Yanes et al. 2010; Maranon et al. 2014). Rats were placed into individual cages above a receiver (RLA‐3000) and allowed 2 weeks of recovery. Following the recovery period, mean arterial pressure (MAP) was measured for 4 days as a baseline period. MAP measurements were obtained during a 10‐second sampling period (500 Hz), recorded and averaged every 5 min for 24 h per day. Rats were then given losartan (40 mg/kg/day) in drinking water for 6 days, and MAP measured 24 h per day as described. Water intake was measured daily and was approximately 30 ml/day for both groups.

### Renal norepinephrine measurements

To make certain that the kidneys were denervated, renal norepinephrine content was measured by LC‐MS by David Mattson, Ph.D., at the Medical College of Wisconsin, as we previously described (Maranon et al. 2014). Norephinephrine content was factored for kidney weight and expressed as pg/mg wet weight of the kidney.

### Statistical analyses

All data are expressed as mean ± SEM. Data were analyzed by Student's *T* test (for two groups) or two‐way Analysis of Variance (ANOVA) with multiple repeat measures. Differences were considered statistically significant at *P *<* *0.05.

## Results

### Body weights

Body weights were similar in rats subjected to uninephrectomy, renal denervation or sham surgery, and telemetry transmitter implantation prior to the surgery (sham: 262.0 ± 9.8 g; renal denervation: 257.5 ± 10.0 g; *P *= NS). At the end of the study, rat body weights were also similar (sham: 273.8 ± 7.3 g; renal denervation: 256.3 ± 17.2 g; *P *= NS). Left kidney weights were also not different in sham compared to renal denervated rats at the end of the experiment (sham: 1.09 ± 0.03 g; renal denervated: 1.21 ± 0.09 g; *P* = NS).

### Renal norepinephrine Content

**Figure 1:**
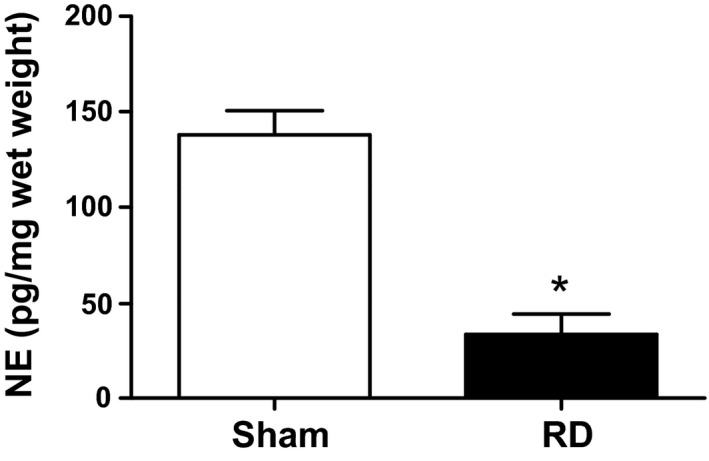
Norepinephrine levels were significantly reduced in rats subjected to renal denervation. At the end of the experiment, kidneys of sham and renal denervated rats (RD) were removed and norepinephrine (NE) levels measured. *P < 0.05 compared to sham.

As shown in Figure 1, norepinephrine content was reduced by 76% in kidneys of renal denervated rats compared to sham controls.

### Blood pressure responses

**Figure 2:**
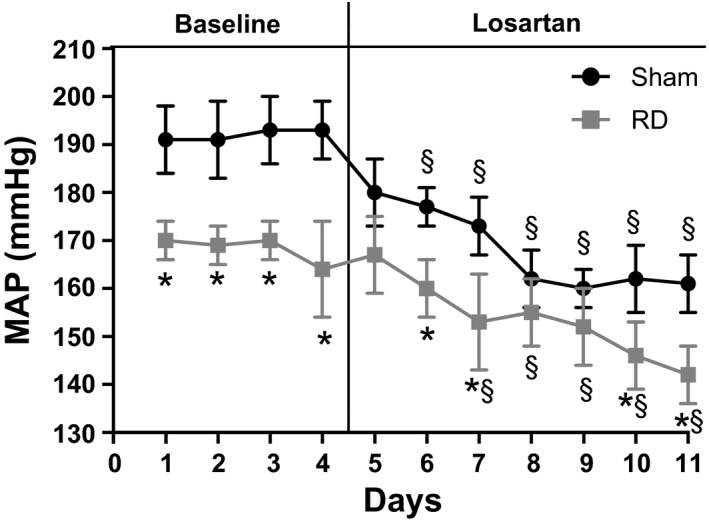
Renal denervation decreased MAP in old female SHR and losartan reduced MAP by similar amounts in both sham and renal denervated rats. MAP was measured by radiotelemetry as described in Methods, for 4 days during a baseline period and for an additional 6 days of losartan treatment. *P < 0.05, sham versus renal denervated rats; ^§^ *P* < 0.05, losartan depressor response compared to baseline MAP in either sham or renal denervated rats.

As shown in Figure 2, renal denervation significantly reduced MAP in old female SHR compared to sham rats. Sham old females had a faster depressor response to losartan than did renal denervated rats, but by the end of day 6 of the losartan treatment period, both the absolute numerical reduction (Sham: 26 ± 4 mm Hg; renal denervated: 26 ± 9 mm Hg, *P *= NS) and the percentage reduction (sham: 14.0 ± 2.3%; renal denervated: 15.2 ± 4.3%; *P *= NS) in MAP with losartan were similar between the groups. By the last 2 days of the losartan treatment period, MAPs were lower in renal denervated rats than sham controls, but MAPs remained above 140 mm Hg for both groups.

## Discussion

This study shows that: (1) renal denervation in old female SHR significantly reduces MAP compared to sham females, as we have previously shown (Maranon et al. 2014); (2) losartan reduces MAP in both renal denervated and sham rats by the same percentage and numerical amount; (3) with the combination of renal denervation and losartan, MAP remains significantly elevated in the old females. These data do not support our hypothesis since we anticipated that renal denervation would attenuate the effect of losartan to reduce blood pressure in our old female SHR. The data suggest then that both the renal sympathetic nervous system and the RAS play distinct, independent roles in mediating the hypertension in old female SHR.

In this study, the combination of renal denervation and losartan failed to reduce the MAP below 140 mm Hg. While we have not measured the blood pressure in old female WKY rats, we have measured the MAP in old female Sprague‐Dawley rats, and found their blood pressures to be approximately 100 ± 4 mm Hg by catheter at 18 months old (Reckelhoff 1997) and 104 ± 3 mm Hg by telemetry at 16 months old (Patil and Reckelhoff, data not published). Gallo and colleagues measured systolic blood pressure in 13 months old WKY females and found it to be 133 ± 4 mm Hg (Gallo et al. 2012). Haloui and colleagues measured MAP in female SHR (160 ± 10 mm Hg) and Brown Norway (110 ± 5 mm Hg) at 80 weeks of age (20 months), and other genetic strains (Haloui et al. 2013). Since these investigators were evaluating the differences in blood pressure among the strains, they considered MAP < 128 mm Hg as the definition for normotension. Thus, because the blood pressure in the old female SHR subjected to renal denervation and losartan in this study remained above 140 mm Hg, we consider the rats to have remained hypertensive. The data suggest then that mechanisms that are independent of both the RAS and the renal sympathetic nervous system also contribute to maintain the hypertension in old female SHR.

In support of this contention, in previous studies, we have found the depressor responses to antagonists of various systems known to contribute to elevated blood pressure are different in aging female SHR. For example, losartan reduced MAP to normotensive levels (≈100 mm Hg) in old male SHR, but not old females (Yanes et al. 2010), as shown in this study where MAP remained 161 ± 9 mm Hg in uninephrectomized female SHR after 6 days of losartan. ET_A_ receptor antagonists failed to reduce MAP in either young females or old males, but reduced MAP in old females, although not to normotensive levels (Yanes et al. 2005; Lima et al. 2013). We recently showed that female SHR have sympathetic activation and adrenergic blockade reduces their blood pressure, but again, the rats remained hypertensive (MAP = 141 ± 7 mm Hg) (Lima et al. 2013). Similarly, renal denervation had a greater effect on MAP in young females than old females (Maranon et al. 2014), supporting the present studies that renal denervation only reduced the MAP in old female SHR by 20 mm Hg to 170 ± 5 mm Hg. Finally, intrarenal microvascular 20‐HETE levels are higher in old females than young females, and blockade of eicosanoid synthesis (1‐aminobenzotriazole) reduces the blood pressure to a greater extent in old females (Yanes et al. 2011). Taken together, with the present studies, the data suggest that the hypertension in old female SHR is mediated by a combination of the RAS, the sympathetic nervous system and the renal nerves, 20‐HETE, and endothelin. This is in stark contrast to old male SHR in which the RAS (Yanes et al. 2010) and the renal sympathetic nerves (Maranon et al. 2014) appear to play the greatest roles in mediating their hypertension with aging.

There are two caveats to our present studies. One is that while the MAP seemed to reach a plateau in the rats treated with losartan alone, it is possible that the MAP may not have reached a plateau by the end of the 6 days in those with renal denervation + losartan. In our previous studies, MAP in old female rats given losartan alone reached a plateau by 6–7 days, whereas MAP in young female SHR continued to decrease throughout the 24‐day losartan treatment period (Yanes et al. 2010). For this reason, the study was designed as it was to allow losartan treatment for 6 days. This was also necessary in order to complete the study before the renal nerves grew back.

The other caveat to our study is that the renal norepinephrine levels were only reduced by 75% in the old females subjected to renal denervation. In other studies in males and in our own in young female SHR (Iliescu et al. 2006), renal denervation reduces norepinephrine levels by 90%. In our previous studies with renal denervation alone in old female SHR, the renal norepinephrine levels were reduced by similar amounts as in this study (Maranon et al. 2014). In other studies in females, Intapad and colleagues found similar reductions in renal norepinephrine content after bilateral renal denervation in controls (213.9 ± 18.9 pg/mg vs. 30.2 ± 7.5 pg/mg protein) and in adult intrauterine growth‐restricted females (182 ± 5.9 vs. 29.9 ± 7.3 pg/mg protein) (Intapad et al. 2013). Although these colleagues are in our university, their renal norepinephrine levels were measured at Vanderbilt and ours were measured at the Medical College of Wisconsin. Thus, we cannot rule out that there may have been some renal nerve regrowth by the time of the end of our study. If this was the case, we would have expected to see the blood pressure increase rather than continue to fall as we did in the renal denervated rats with losartan.

### Perspectives

As mentioned previously, hypertension in aging women has been shown in many studies to be less well controlled than in age‐matched men (National Center for Health Statistics, Health, United States, 2011; Lima et al. 2012). The reasons for this are unknown. Our data suggest that there may be different mechanisms responsible for essential hypertension in men and women and that these mechanisms may change with advancing age. More precisely, our data suggest that similar mechanisms, but to different extents, may be contributing to elevated blood pressure in aging men and women. In addition, it is possible that the efficacy to antihypertensive medications may be different in men and women and only a few studies of this type have been done to date since most of the studies combine the data for men and women and do not evaluate the genders separately. In this age of individualized, personalized medicine, the guidelines for antihypertensive treatment should be re‐evaluated and studies done with these concepts and questions in mind.

## Conflict of Interest

None declared.
